# Blue Bounty: Italy’s Dual-Use Solution for Crab Invasion, Nutritional Value, Safety, and Valorization

**DOI:** 10.3390/toxics12070506

**Published:** 2024-07-14

**Authors:** Eleonora Di Salvo, Antonino Nazareno Virga, Salvatore Forgia, Luca Nalbone, Claudia Genovese, Vincenzo Nava, Concetto Mario Giorgianni, Rossella Vadalà, Nicola Cicero

**Affiliations:** 1 Department of Biomedical, Dental, Morphological, and Functional Image Sciences (BIOMORF), University of Messina, 98100 Messina, Italy; edisalvo@unime.it (E.D.S.); vincenzo.nava@unime.it (V.N.); rossella.vadala@unime.it (R.V.); 2 Department of Agricultural, Food and Forestry Sciences, University of Palermo, 90121 Palermo, Italy; antoninonazareno.virga@unipa.it; 3 Department of Veterinary Sciences, University of Messina, Polo Universitario dell’Annunziata, Viale Giovanni Palatucci SNC, 98168 Messina, Italy; salvatore.forgia@studenti.unime.it (S.F.); luca.nalbone@unime.it (L.N.); 4 National Research Council, Institute for Agriculture and Forestry Systems in the Mediterranean, 95128 Catania, Italy; claudia.genovese@cnr.it; 5 Department of Biomedical Sciences, Policlinico Universitario Messina, University of Messina, 98124 Messina, Italy; concetto.giorgianni@unime.it; 6 Science4life Spin-Off Company, University of Messina, 98168 Messina, Italy

**Keywords:** *Callinectes sapidus*, blue crab, non-indigenous species, fatty acid profiles, GC-FID analysis, microbiological analysis, microplastics

## Abstract

In the past few years, non-native blue crab has been expanding its range in the Adriatic and Mediterranean Seas. However, when non-indigenous species establish a lasting presence in a novel ecosystem and actively expand into other regions, posing potential risks to local biodiversity and economic harm, they are classified as invasive species. For this study, two different batches of blue crabs were collected: one from the Mediterranean Sea and the other from the Adriatic coast area. Considering the negative ecological impact that blue crab has had and continues to have on the Italian coasts, this work was aimed to evaluate the characteristics of the Adriatic Sea “variant”; another goal was to propose the potential use of blue crab as a commercially profitable source due to its organoleptic characteristics. Data obtained revealed a high protein content, a good lipid profile, a low number of plastic particles, and the absence of toxic metals and pathogenic microorganisms. A deep statistical analysis was conducted to compare different portions of blue crab from the Adriatic and Mediterranean Seas. The results suggest that *Callinectes sapidus* is beneficial for human consumption and represents a valuable seafood, and its carapace could be used as compliant feed for aquaculture due to its metal composition and microbiological content.

## 1. Introduction

Blue crab (*Callinectes sapidus*) is a member of the Portunidae family of decapod crustaceans and is native to the Atlantic coasts of the Americas. It is commonly found in estuarine environments and has a broad distribution, ranging from South America and the Caribbean to the Gulf of Mexico and from the eastern seaboard of North America to the north of New England. Within this extensive range, the blue crab plays a crucial role in estuarine food webs, functioning as a dominant benthic predator and scavenger with opportunistic behavior [1]. Apart from its ecological significance, blue crabs play a crucial role in sustaining both commercial and recreational fishing industries across a substantial portion of their habitat. In the United States, commercial fisheries operate along the coastal region from Texas to New York, with major catches coming from Maryland, Virginia, North Carolina, and Louisiana [2]. In the past few years, there has been a notable migration of blue crabs. Blue crab species were unintentionally introduced to the Mediterranean region in 1948 in Greece, as documented by Zenetos et al. [3]. Subsequently, their populations have steadily grown, presenting a potential threat to indigenous fisheries and the overall biodiversity. The increase in abundance could be due to climate change [4] or to the continued introduction of *C. sapidus* from North America via fishing vessels [5]. However, when non-indigenous species establish a lasting presence in a novel ecosystem and actively expand into other regions, posing potential risks to local biodiversity and economic harm, they are classified as invasive species. Additionally, they can contribute to the increased prevalence of new diseases and exert detrimental impacts on the economic well-being of colonized areas. Consequently, they are recognized as a primary factor contributing to the decline in biodiversity. Notably, crustaceans exemplify this phenomenon, with the Atlantic blue crab (*C. sapidus*) [6] serving as a prominent example. Due to the physiological traits and remarkable adaptability of blue crab, which enable it to thrive across various temperatures and salinities, this species has successfully established itself in European seas [6]. Furthermore, the absence of natural predators in these waters allows blue crabs to flourish, as they feed on valuable species such as bivalves, followed by fish, shrimps, and gastropods, thereby impacting coastal marine trade through a combination of consumption and potential ecological disruption. In particular, the Mediterranean and Adriatic Seas [7,8] have documented adverse effects on native biodiversity and regional economies. Despite the limited information on the ecological consequences of species in invaded habitats, these negative interactions have been noted [9,10]. Individual surveys have indicated a decline in fishermen’s income, with estimates ranging from 15% to 70%, following the appearance of blue crabs in the seas, which have been observed to cause damage to fishing nets and catches, representing a significant economic loss [11]. Moreover, in the last 20 years, the intensification of pollution has been noticed. Specifically, marine ecosystems are experiencing increasingly significant transformations because of pollutants generated by human activities, including heavy metals, pesticides, plastics, and microplastics [12]. The current understanding reveals that crabs tend to amass microplastics in different parts of their bodies, including the stomach, hepatopancreas, tissues, and breast chest [13]. The present research evaluates the potential of *Callinectes sapidus*, caught off the Italian coast, as a viable food source for human consumption and as aquaculture animal feed. Furthermore, other authors have reported that all edible tissues of blue crab exhibit a high protein content (>23%), with muscle fat content varying between 2% and 3.69% of the wet weight. Additionally, its lipid content is >5%, making blue crab an appropriate food choice for those following a low-fat diet. Furthermore, all nutritional indices have been shown to be beneficial to human health [11]. Comprehensive chemical and microbiological analyses were conducted, as well as the detection of microplastics. Subsequently, the nutritional value of edible blue crab tissues, including crab meat and claw meat, and nonedible tissues was investigated. The final purpose of this work is to promote the consumption of blue crab in Italy and to determine the suitability of incorporating blue crab waste into animal feed for aquaculture.

## 2. Materials and Methods

### 2.1. Crab Sampling and Sample Preparation

**Figure 1:**
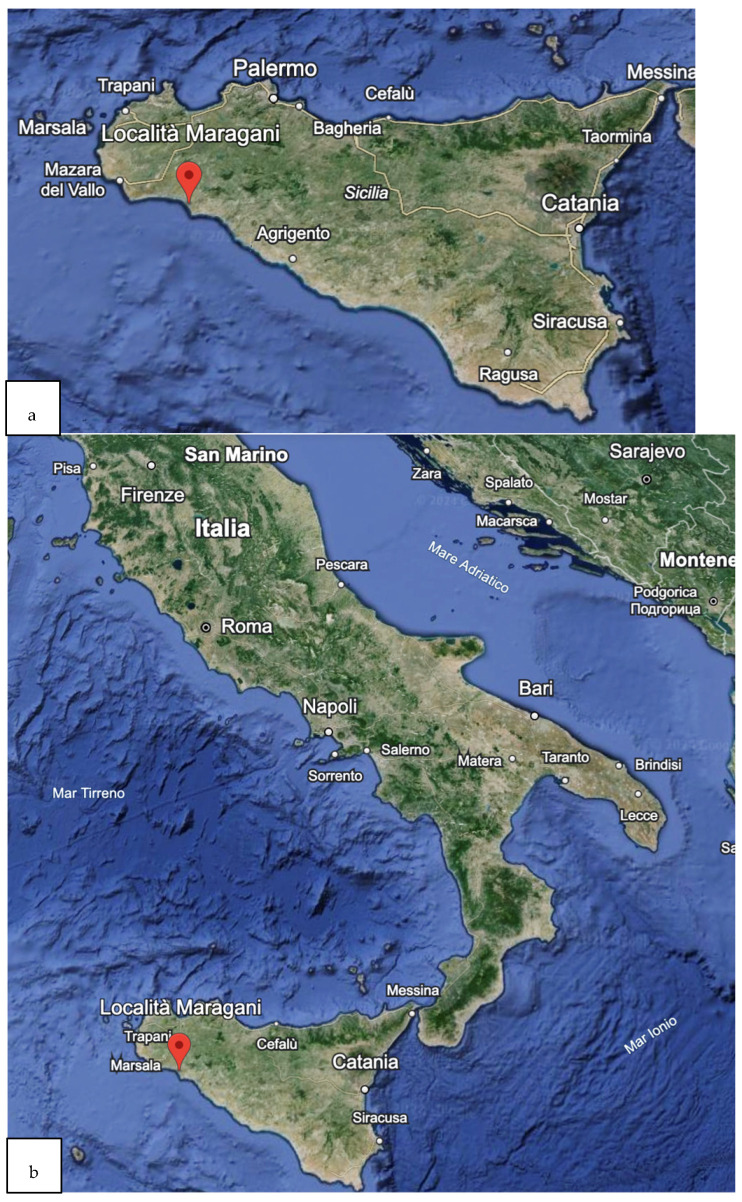
(**a**). Site position of the Maragani River in Sicily, Italy (Mediterranean Sea). (**b**) The Adriatic coast area (Italy) for the catch of *Callinectes sapidus* used in the experiment.

The present study employed two distinct batches of blue crabs sourced from local markets. The first was obtained from the Mediterranean Sea, situated near the Maragani River (Agrigento, Sicily) (Figure 1a), while the second was procured from the Adriatic coast area (FAO subarea 37.2.1) (Figure 1b). Both batches were collected in November 2023. The *C. sapidus* samples were then transported to the laboratory of the University of Messina, Italy. Once at the laboratory, the crabs were divided into three subsamples: the first for chemical composition analysis, the second for microbiological analysis, and the third for microplastic detection.

Chemical characterization was performed on eight male adult blue crab samples, four from the Mediterranean Sea and four from the Adriatic Sea. The samples were analyzed in three distinct portions, the body meat (crab meat), pulp of the claw (claw meat), and carapace, to investigate their diverse chemical compositions. 

For microbiological characterization (n. 10) and microplastic detection (n. 10), only blue crabs from the Adriatic coast area were analyzed, since samples from the Maragani river were insufficient.

### 2.2. Chemical Analysis

#### 2.2.1. Chemicals and Reagents

For protein analysis, the Kjeldahl catalyst was supplied by Carlo Erba (Milan, Italy). Analytical-grade solvents and chemicals used for proximate composition analysis, lipid extraction, and fatty acid analysis were purchased from Merck (Darmstadt, Germany). A mixture of fatty acid methyl esters (Supelco 37-Component FAME Mix) useful for fatty acid analysis was purchased from Sigma-Aldrich (Darmstadt, Germany). For the screening of mineral elements, reagents such as HNO_3_ (65% *v*/*v*) and H_2_O_2_ (30% *v*/*v*), which were used for mineralization, were of Suprapur grade (Mallinckrodt Baker, Milan, Italy). Ultrapure water (<5 mg/L TOC), which is essential for sample dilution after acid digestion, was obtained from a Barnstead Smart2Pure 12 water purification system (Thermo Scientific, Milan, Italy). Single-element standard solutions of Al, As, Be, Ca, Cd, Co, Cr, Cu, Fe, Li, Mg, Mn, Mo, Na, Ni, P, Pb, Sb, Se, Ti, V, and Zn (1000 mg/L each in 2% HNO_3_); on-line internal standard solutions of ^45^Sc, ^73^Ge, ^115^In, and ^209^Bi (1000 mg/L in 2% HNO_3_); and the internal standard solution of Re (1000 mg/L in 2% HNO_3_) were obtained from Fluka (Milan, Italy). For mercury analysis, Hg solution (1000 mg/L in 3% HCl) and pure HCl (37%) were obtained from Merck (Darmstadt, Germany). Certified matrix ERM-CE278k Mussel Tissue was purchased from Merck Spa, Sigma-Aldrich, Milan, Italy.

#### 2.2.2. Proximate Composition

Blue crab samples were subjected to triplicate analysis to determine their proximate composition. Protein and ash contents were evaluated in accordance with the AOAC Official Methods 990.03 and 923.03 (2005) [14], respectively. Residues were analyzed for protein content using Kjeldahl methods, with a factor of 6.25 employed to calculate the protein content in milligrams. For the ash analysis, 1 g of the sample was incinerated at 525 °C for 5 h. The moisture content (MC) was calculated as follows:

MC (%)=(P2−P3)÷(P2−P1)×100

Here, ***P*1** is the weight of the vessel with the lid, ***P*2** represents the weight of the vessel with the lid and the sample that was previously dried, and ***P*3** corresponds to the weight of the vessel with the lid and the sample after drying.

#### 2.2.3. Lipid Extraction and Preparation of FAMEs

Lipid fraction extraction was conducted using a modified version of the Folch method, a technique for isolating and purifying total lipids from animal tissues. The entire contents of each product were homogenized, and 4 g of the homogenates were transferred into 50 mL tubes. Folch’s solution (chloroform: methanol, 2:1, *v*/*v*) was added to the tubes, followed by a 0.73% NaCl solution. The samples were vortexed for 1–2 min and then centrifuged at 3500 rpm for 15 min at 4 °C. The lipid fraction (bottom layer) was collected in a preweighed flask and dried using a rotating evaporator (Heidolph Instruments GmbH & Co., Schwabach, Germany). The total lipid contents (g/100 g) were determined through gravimetry. Fatty acid methyl esters (FAMEs) were obtained by transmethylation (hot esterification) of the lipid fractions of the analyzed samples. This process involved adding a methanol–sulfuric acid mixture (9:1, *v*/*v*), following the ISO 5509:2000 method [15]. The mixture was heated in an oven at 100 °C for 1 h, with butylated hydroxytoluene (BHT) serving as an antioxidant to protect polyunsaturated fatty acids (PUFAs) from high temperatures. The supernatant was diluted with n-hexane after collection.

#### 2.2.4. GC-FID Analysis

The fatty acid composition of the samples was examined using a gas chromatograph equipped with a split/splitless injector and flame ionization detector (GC-FID, Dani Master GC, Dani Instrument, Milan, Italy). The instrumental analyses followed the procedure summarized by Nava et al. [16]. Data acquisition and management were facilitated using Clarity Chromatography v4.0.2 software (DataApex, Prague, Czech Republic). Each sample underwent triplicate analysis, including analytical blanks.

#### 2.2.5. Atherogenicity Index (AI) and Thrombogenicity Index (TI)

To assess and provide information on the health and nutritional potential of lipids in the analyzed samples, the atherogenicity index (AI) and thrombogenicity index (TI) were evaluated. The atherogenicity index expresses the ratio between the sum of the main saturated fatty acids and the sum of the main classes of unsaturated fatty acids. The former are considered proatherogenic because they promote the adhesion of lipids to the cells of the immunocirculatory system. The latter are considered anti-atherogenic because they inhibit plaque aggregation and reduce the levels of esterified fatty acids, cholesterol, and phospholipids, thus playing a preventive role in the development of coronary micro- and macropathologies [17,18]. The following equation was used to calculate AI:

AI = [C12:0 + (4 × C14:0) + C16:0]/[Σ n-6 PUFA + Σ MUFA + Σ n-3 PUFA]

The thrombogenicity index indicates the probability of blood clot formation in blood vessels. It was derived by comparing prothrombogenic fatty acids (SFA) with antithrombogenic fatty acids (MUFA, n-6 PUFA, and n-3 PUFA) [17,19].

TI was calculated using the following equation:

TI = [C14:0 + C16:0 + C18:0]/[0.5 × Σ n-6 PUFA + 0.5 × Σ MUFA + 3 × Σ n-3 PUFA + (n-3 PUFA/n-6 PUFA)]

#### 2.2.6. Flesh-Lipid Quality Index

The flesh-lipid quality (FLQ) index quantifies the percentage correlation between the main n-3 PUFAs (EPA + DHA) and total fatty acids. Higher values of this index indicate a higher quality of the dietary lipid source [20].

The expression used to calculate the FLQ index is given below:

FLQ = 100 × [EPA + DHA]/[% of total fatty acids]

#### 2.2.7. Elemental Analysis

To determine mineral element content, samples underwent a pretreatment step involving acid digestion in a closed-vessel microwave digestion system (ETHOS 1, Milestone, Bergamo, Italy). The mineralization procedures varied based on the specific part of the crab under consideration and followed those reported by Tardugno et al. [21]. For the edible portion, approximately 0.5 g of each sample was precisely weighed in PTFE vessels. These were then fortified with 1 mL of internal standard Re at a concentration of 0.5 mg/L and mixed with 7 mL of HNO_3_ and 1 mL of H_2_O_2_. The digestion program was performed under the following conditions: 15 min from room temperature to 180 °C (step 1) and 15 min at a constant 180 °C (step 2), both at a microwave power of 1000 W. Subsequently, 1 mL of Re, an internal standard (at 0.5 mg/L), along with 6 mL of HCl and 2 mL of HNO_3_ were added to approximately 0.5 g of each carapace sample. The operating conditions were 10 min at a temperature ranging from 0 °C to 180 °C (step 1) and 10 min at a constant temperature of 200 °C with a constant microwave power of 1000 W. In each pretreatment method, the third step involved a 20 min cooling phase. Following pretreatment, samples were diluted to a volume of 25 mL with ultrapure water and filtered through 0.45 μm PTFE filters. Both the blank solution (HNO_3_ and H_2_O_2_ in a 7:1 *v*/*v* ratio for the edible part, HCl and HNO_3_ in a 6:2 *v*/*v* ratio for the carapace) and the certified reference material were prepared under the same conditions as the samples.

#### 2.2.8. ICP-MS Analysis

Analysis using a single-quadrupole inductively coupled plasma-mass spectrometer (ICP-MS, iCAP-Q, Thermo Scientific, Waltham, MA, USA) permitted the determination of the following elements: Al, As, Be, Ca, Cd, Co, Cr, Cu, Fe, Li, Mg, Mn, Mo, Na, Ni, P, Pb, Sb, Se, Ti, V, and Zn. The operative conditions were the same as those reported by Di Salvo et al. [22] and Lo Turco et al. [23]. All samples were analyzed in triplicate, along with the analytical blanks. The mercury (Hg) levels were quantified using a direct mercury analyzer (DMA-80) manufactured by Milestone S.r.l., Sorisole (BG), Italy. The analysis was performed according to the EPA method 7473 [24]. The procedure was conducted in accordance with the methodology described by Nava et al. and Graci et al. [25,26], with some modifications. 

### 2.3. Microbiological Analysis

A total of 10 crabs were processed for microbiological characterization. The carapaces and claws were rinsed with 70% ethanol and opened using sterile forceps. The abdominal and claw pulps were separately processed as separate samples. The pulp from each district was collected aseptically and divided into three aliquots. The initial aliquot was employed for the enumeration of *Enterobacteriaceae*, according to ISO 21528-2:2017 [27], on plates of Violet Red Bile Glucose Agar (Biolife, Milano, Italy) incubated at 37 ± 1 °C for 24 ± 2 h; the second aliquot was utilized for the count of specific spoilage organisms (SSOs) on Lyngby Iron Agar (Oxoid Ltd., Basingstoke, UK) incubated at 20 °C for 72 ± 3 h distinguishing between non-sulfide-producing bacteria (white colonies) and sulfide-producing bacteria (black colonies) [28]; and the third aliquot was used for the detection of *Salmonella* spp. according to ISO 6579-1:2017 [29], with enrichments in Mueller Kauffmann Tetrathionate Broth Base (Biolife, Milano, Italy) and Rappaport Vassiliadis Soy Broth (Biolife, Milano, Italy), followed by a smear on plates of Chromogenic Salmonella Agar (Biolife, Milano, Italy) and Xylose Lysine Deoxycholate Agar (Biolife, Milano, Italy), both incubated at 37 ± 1 °C for 24 ± 3 h.

The second aliquot was instead used for the detection of pathogenic *Vibrio parahaemolyticus* and *Vibrio cholerae* according to ISO/TS 21872-1: 2007 [30].

The third aliquot was diluted with Half Fraser Broth Base (Biolife, Milano, Italy) at a ratio of 1:9 *w*/*v*, homogenized with a stomacher, and processed for the detection of *Listeria monocytogenes*, according to ISO 11290-1:2017 [31], on plates of Agar Listeria, according to Ottaviani and Agosti (Biolife, Milano, Italy), and Listeria Palcam Agar (Biolife, Milano, Italy) and incubated at 37 ± 1 °C for 48 ± 2 h.

### 2.4. Analysis of Microplastics

#### 2.4.1. Sample Preparation and Digestion

A total of 10 blue crabs were processed during 5 separate analysis sessions (2 crabs per session) for the detection of potential microplastics (items) and n. 4 crabs were analyzed for the recovery rate. The carapace of each crab was rinsed with 70% ethanol and opened using forceps. The stomach and hepatopancreas were gently removed, placed into a 1 L conical glass flask, and covered with aluminum foil. According to the protocol proposed by Thiele et al. [32], the stomach and hepatopancreas were digested using 10% potassium hydroxide (Sigma-Aldrich, USA) at a ratio of 1:40 *w*/*v* for 48 h under constant stirring at 40 °C inside an oscillating incubator (Vdrl Asal 711/CT, Bioltecnical Service, Anzio, Italy). After digestion, density separation was performed by adding an equal volume of saturated saline solution. After 24 h, the supernatant was collected using a glass pipette and filtered through a white mixed cellulose ester filter (8 μm pore size and 47 mm diameter; Biosigma, Cona, Italy) using a vacuum glass system (Biosigma, Cona, Italy). Each filter was subsequently placed in a covered 90 mm Ø glass Petri dish (Biosigma, Cona, Italy) and air-dried for several minutes. Once sufficiently dried, each sample and procedural blank filter were examined under a stereomicroscope (Leica, Germany) to identify and enumerate items that could be microplastics (MPs) based on their physical characteristics, following the instructions developed by Hidalgo-Ruz et al. [33]: items < 5 mm in size, uniform color, and equal thickness throughout the length (if fiber) and that, if touched with a needle, did not disintegrate. The visually identified items were then sorted according to shape based on the classification proposed by GESAMP [34].

#### 2.4.2. Polymer Identification of Microplastics

The items detected in the sample filters were analyzed to determine their constituent polymers using Fourier transform infrared (FT-IR) spectroscopy. The selected items were first spotted under a stereomicroscope and then analyzed directly on the filter surface. Fourier transform infrared (FT-IR) analysis involved an IR Affinity-1 spectrometer (Shimadzu, Japan) in single-reflectance ATR mode. Each FT-IR spectrum involved 45 co-scans, measured within the wavelength range of 400 to 4000 cm^−1^ with a resolution of 4 cm^−1^. Particle identification was achieved by comparing the FT-IR absorbance spectra of the analyzed items to those in a polymer reference library, with a minimum acceptable match index of 70%.

#### 2.4.3. Quality Assurance and Quality Control

The samples were processed in a controlled-access room, and all materials, equipment, and laboratory surfaces were thoroughly washed and rinsed with double-filtered deionized water obtained using sterile syringe filters (cellulose acetate) with a pore size of 0.22 μm (VWR International, USA). Sample processing occurred within a clean airflow cabinet, and a procedural blank (consisting of the whole procedure without crabs) was created during each analysis session to assess external contamination, particularly from fibers [35]. The samples were covered with aluminum foil and were generally exposed to air only when necessary and for the shortest possible period. Stereomicroscope observations were performed outside the clean airflow cabinet, with two blank filters placed alongside the samples during analysis to monitor airborne contamination. Any items on the blank filters were compared to those on the sample filters based on color and morphology, and if found to be too similar, items in the sample filters were excluded from the final count. To determine the minimum detectable particle size and recovery rates of the protocol used, various size ranges (40–48 μm, 63–90 μm, 91–125 μm, 126–180 μm, 181–355 μm, and 356–510 μm) of white low-density polyethylene (LDPE) particles (0.924 g/cm^3^) (Sigma-Aldrich, St. Louis, MI, USA) were individually spiked into crab samples. The minimum detectable particle size was determined by spiking 20 individual LDPE particles of a given size range onto crab samples that were processed as described above. The recovery rate was evaluated by individually spiking 20 LDPE particles of the minimum particle size into n. 4 crab samples processed as previously described. Particle recoveries were determined by enumerating the number of retrieved particles relative to the amount of added particles.

### 2.5. Data Analysis

The biometric parameters “body weight”, “carapace width”, and “carapace length”, were recorded to evaluate possible correlations with MP abundance using simple linear regression analysis. The term “item” is used throughout the text to refer to particles that, by their aspect, may appear to be microplastics but have not been chemically characterized, so their actual polymer composition is unknown. The normal distribution of the data was tested using the D’Agostino–Pearson omnibus test. Additionally, any significant differences between the microbial loads of the abdominal and claw pulp, as well as between the crabs, were analyzed using the Mann–Whitney non-parametric test. Data are expressed as the mean ± standard deviation (s.d.) of triplicate measurements. Significant differences (*p* < 0.05) within means were analyzed by two-way ANOVA followed by Tukey’s honestly significant difference (HSD) using the XLStat statistical 14 software Microsoft Excel data analysis add-on (Microsoft Corporation, Redmond, WA, USA).

## 3. Results

### 3.1. Blue Crab Chemical Characteristics

As shown in Table 1, the protein and total fat levels varied notably between the blue crabs sourced from two distinct marine environments. The maximum moisture content (78.22%) was found in Adriatic blue crab meat and the minimum in Mediterranean crab meat (78.16%). The protein content was significantly higher in claw meat from both the Adriatic and Mediterranean Seas. The statistical analysis revealed that the composition of crab meat in terms of moisture, protein, ash, and total fat content varied significantly based on the sample portion and sampling area. Furthermore, a significant interaction was observed between the protein and total fat and sampling areas, suggesting that the effect of the area of origin on crab meat composition varies depending on the sampling area. These findings indicate that the selection of the sampling portion and sampling area can have a significant impact on the nutritional characteristics of crab meat. The results were consistent with those previously reported by other authors [36].

### 3.2. Mineral Contents

In this study, 23 mineral elements (Al, As, Be, Ca, Cd, Co, Cr, Cu, Fe, Hg, Li, Na, Mg, Mn, Mo, Na, Ni, P, Pb, Sb, Se, Ti, V, Zn) were analyzed in *C. sapidus* specimens caught in two different Italian Seas (Adriatic and Mediterranean) using ICP-MS and DMA-80 analysis. In addition, the methods were previously validated, and good results were obtained. In fact, linearity was optimal with R^2^ > 0.9988; the sensitivity was very good, with LOD values ranging from 0.001 to 0.107 mg/kg, while LOQs ranged from 0.003 to 0.353 mg/kg. Finally, good percentage recovery values were obtained, with a range between 91.46% and 101.75%. The lowest and highest average recovery rates were observed for Na (91.46%) and Cd (101.75%).

Table 2 lists the mineral compositions of the samples. The mineral elements are listed alphabetically, and all values are expressed as mg/kg.

The arsenic levels were similar in all sample types, ranging between 0.05 ± 0.01 and 0.10 ± 0.02 mg/kg. The arsenic content did not reach significant levels.

The toxic element Cd was present in small quantities. The crab meat of *C. sapidus* from both Seas contained a higher concentration of cadmium, and the difference was significant (*p* < 0.05). The Cd concentration in this study was found to be significantly lower than the limit imposed by the European Food Safety Authority (EFSA) and also lower than the results of other studies [37,38]. The concentration ranged from 0.06 ± 0.01 to 0.13 ± 0.01 mg/kg.

Cr concentrations were slightly higher in crab meat (0.12 ± 0.02–0.13 ± 0.01 mg/kg) but much lower in claw meat (0.08 ± 0.01 mg/kg–0.07 ± 0.01 mg/kg) and undetectable in carapace samples. The Cr concentration detected in this study was slightly higher than that reported by other authors [39].

Cu and Fe levels varied but were significantly higher in all parts of *C. sapidus*, in accordance with other findings [39].

The highest mercury content was obtained for claw meat samples from the Mediterranean Sea (0.11 ± 0.02 mg/kg), with no significant value observed for other samples.

Ni had the highest concentration in crab meat (0.19 ± 0.02–0.21 ± 0.04 mg/kg). For the other sample parts, the Ni content was much lower, below the LOQ for the carapace in the Adriatic Sea. The Ni value was in line with the results reported by Hosseini et al. [38]. Pb levels in crab meat ranged from 0.19 ± 0.03 to 0.21 ± 0.02 mg/kg, with values slightly higher in claw meat (0.23 ± 0.05–0.25 ± 0.03 mg/kg), and below the LOQ in carapace samples. The results obtained in this study were found to be lower than those reported by other authors [34] and significantly lower than the limits reported by the EFSA for Pb (0.50 mg/kg). Se content was slightly higher, especially in crab meat samples (151.29 ± 1.98–156.38 ± 5.33 mg/kg) from the investigated blue crabs in the Adriatic and Mediterranean Seas. Carapace samples showed lower selenium concentrations (33.23 ± 1.87–35.66 ± 0.47 mg/kg).

Be, Li, Mo, Sb, Ti, and V were below the LOQ in all blue crab samples.

In summary, the aluminum, sodium, calcium, phosphorus, selenium, copper, iron, nickel, cobalt, lead, manganese, and zinc values for blue crab were significantly higher (*p* > 0.05), with no particular differences between *C. sapidus* caught in the Adriatic and Mediterranean Seas. Sodium was the most abundant mineral present in all blue crab samples. Cadmium, nickel, arsenic, and chromium were present in trace amounts in all samples except the carapace samples. Calcium values were found to be significantly higher (*p* < 0.05) in all parts of the blue crab. The concentration of mineral elements classified as toxic by the EFSA did not exceed the legal limits (Commission Regulation (EU) 2023/915).

### 3.3. Fatty Acid Composition

The results showed that the fatty acid profiles were significantly different between the claw and crab meat and the carapace of blue crab. The results are reported in Table 3 and Table 4.

In general, the most abundant fatty acids discovered in all samples were palmitic acid (C16:0), stearic acid (C18:0), oleic acid (C18:1 n-9), linoleic acid (C 18:2 cis), palmitoleic acid (C 16:1 n-7), eicosapentaenoic acid (EPA) (C 20:5 n-3), and docosahexaenoic acid (DHA) (C22:6 n-3). 

The percentage of total saturated fatty acids (SFAs) ranged from 28.48% to 30.09% in Mediterranean Sea samples and from 28.72% to 29.80% in Adriatic Sea samples. The total of n6 fatty acids were 6.59% ± 0.08, 6.89% ± 0.03, and 6.08% ± 0.07 in the Adriatic Sea samples of crab meat, claw meat, and carapace, respectively. The total n6 fatty acids were 6.77%± 0.08, 7.04% ± 0.05, and 6.07% ± 0.05 for crab meat, claw meat, and carapace samples, respectively, in the Mediterranean Sea. The content of arachidonic acid (20:4n6), which becomes desaturated and elongated for the formation of long-chain fatty acids, was highest in all analyzed samples from both seas.

The total amount of n3 was considerably higher in all samples of Adriatic and Mediterranean Seas. The ratio n3/n6 was slightly higher in the carapace samples of blue crabs from both seas, as reported by other authors [40]. This ratio is important for human health.

The total EPA (C20:5n3) contents were significantly higher, at 17.35% ± 0.04 in the Mediterranean Sea and 17.24 ± 0.03 in the Adriatic Sea; while DHA (C22:6n3) concentration were higher (12.17%± 0.03 and 11.66%± 0.04) in crab meat from the Mediterranean Sea and in claw meat from the Adriatic Sea. Some researchers reported that the concentrations of EPA ad DHA and the (n3/n6) ratio could change based on age, sex, tissues, and species of blue crab and the characteristics of the sea (salinity, temperature, pH) [41,42]. The flesh-lipid quality index (FLQ) indicates a higher-quality dietary lipid source. As a result, the FLQ was lower in the *C. sapidus* crab meat.

The total polyunsaturated fatty acid (PUFA) composition was higher, especially in the crab meat of the Mediterranean Sea samples and in the claw meat of the Adriatic Sea samples. As expected, the total monounsaturated fatty acid (MUFA) content was higher in crab meat and carapace samples from the Adriatic Sea and in crab meat and carapace sample from Mediterranean Sea samples. 

Table 3 and Table 4 present a comparison of the atherogenicity index (AI) and thrombogenicity index (TI) established in the analyzed samples. In particular, high AI and TI values could be indicators of thrombus formation and platelet aggregation, whereas lower values of these indicators may be considered wellness indicators for human health [17]. In this study, all samples analyzed are indicated as means ± standard deviations for the AI value and a TI value of less than one.

The findings found in this study, when compared with those from other authors studying the same species of blue crab, show significant differences in fatty acid composition [40,43]. These differences seem to be linked to inter-species variations, age, sex, dietary nutrient content, environmental conditions, seasonal fluctuations, taste profiles, and additional quality attributes of crab-derived products.

### 3.4. Microbiology

In all the analyzed samples, *L. monocytogenes*, *Salmonella* spp., *V. parahaemolyticus*, and *V. cholerae* were not detected, whereas *Enterobacteriaceae* levels were below the limit of detection (LOD 10 CFU/g). Regarding SSOs, an average load of 3.61 ± 0.54 log CFU/g, ranging from 2.85 to 5.02 log CFU/g, was observed. In detail, an average load of 3.88 ± 0.57 log CFU/g (range: 3.15–5.02 log CFU/g) was observed for abdominal samples, whereas an average load of 3.34 ± 0.36 log CFU/g (range: 2.85–3.82 log CFU/g) was detected for claw samples. No significant differences were observed between the average SSO loads of the abdominal and claw samples (*p* > 0.05).

The average SSO loads for individual crabs were 3.73 ± 0.65 log CFU/g and 3.49 ± 0.39 log CFU/g, respectively. No significant differences were observed between the average SSO loads of the blue crabs samples (*p* > 0.05).

Sulfide-producer SSOs (black colonies) were detected only in n. 3 (15%) abdominal samples with an average load of 2.74 ± 1.26 log CFU/g collected from crabs.

### 3.5. Microplastic Detection

A total of eight items were detected in the stomach and hepatopancreas of six crabs (60%) with an average of 0.8 ± 0.79 items/individual and 0.003 ± 0.004 items/g. A maximum of two items per sample and 0.008 items/g were observed.

In terms of shape, the only types observed were fibers, while in terms of color, seven represented blue and one was translucent. For the size, six items (75%) ranged between ~100 µm and ~500 µm, one between ~501 µm and ~1000 µm, and one between ~1001 µm and ~2000 µm.

Out of the eight visually identified items, six items were chemically identified as cotton, while two items detected from two different crabs were confirmed to be polyester MPs by FT-IR analysis. Therefore, considering the identified MPs, the positive sample was 20%. 

Regarding procedural blank and blank filters, the average amounts of items detected were 0.20 ± 0.50 fiber/filter and 0.40 ± 0.58 fiber/filter, respectively. Most contaminations were represented by black fiber >~1000 μm in length.

Among the different size classes tested, the 40-48 μm range was the smallest in which LDPE particles could be easily detected under a stereomicroscope. A total of 75 LDPE particles were counted on the filters of the four spiked crab samples, with a recovery rate of 93.75%. Regarding the biometric parameters, an average body weight of 213.71 ± 24.51 g, an average carapace width of 14.65 ± 1.54 cm, and an average carapace length of 7.1 ± 0.7 cm were determined for the crabs herein analyzed for MP detection. No significant correlation was observed between item/MP abundance and biometric parameters.

## 4. Discussion

This study focused on chemical and microbiological analysis, aiming to determine whether *Callinectes sapidus* can be included in new Italian cuisine and whether its carapace can serve as aquaculture feed. The moisture content was found to be significantly higher in crab meat from both seas, with values of 78.22 ± 0.05 and 78.16 ± 0.10, respectively. A second study yielded comparable results, with a moisture value range from 79.050 ± 0.07 to 78.020 ± 0.03 [40]. Protein content differed between the various regions and between the two seas. Only the protein content in claw meat from both seas was higher than that reported in a previous study [44]. The percentage of total fat in crab meat from the Adriatic Sea (4.47%) and the Mediterranean Sea (6.21%) was found to be higher than that in the other parts. Küçükgülmez et al. [44] reported an average fat content of 0.440 g/100 g for the breast and claw meat of *C. sapidus*. It was found that the ash content exhibited considerable variation among the considered body parts. The crab meat portion exhibited the highest ash content (1.62 ± 0.05; 1.55 ± 0.03) and was also lower than previously reported. 

In summary, the results were consistent with previous studies [36,44].

Generally, crustaceans inhabiting sediment accumulate heavy metals in their tissues, muscles, and carapaces [45]. Consequently, metal concentrations varied significantly among the three parts of the blue crab. Of course, some metals, such as Cu, Cr, Ni, and Zn, are toxic only at high concentrations. These findings revealed elevated concentrations of Cu and Zn; Ni and Cr were detected in all samples but were not detectable in the carapace samples. The distribution of Cu, Cr, and Ni in blue crab meat indicates that crab meat is the primary tissue for metal accumulation, whereas Zn is mainly found in carapaces. Additionally, nonessential and toxic metals (As, Cd, Pb, and Hg) do not play a metabolic role in crustaceans, and their tissue contents exceed legal limits. Consistently, the results for As, Cd, and Pb were significantly positive (*p* < 0.05), except in the carapace samples. However, the permissible limits of Cd and Pb in shellfish are 3 and 1.5, respectively [46]. In addition, the mercury (Hg) levels were very low in all samples analyzed. EU Commission Regulation 2023/915 [47] sets a maximum limit for Cd, Pb, and Hg in crustaceans of 0.50 mg/kg; thus, the measured concentrations of these metals were lower than the EU regulation. Overall, the results demonstrate a relatively low concentration of toxic elements, including arsenic, lead, and chromium, compared with other studies [45]. On the other hand, the concentration of essential elements, such as magnesium, was notably higher. 

Fatty acid profiles differed among the crab meat, claw meat, and carapace of the blue crab and were also diverse between the blue crabs caught in the Adriatic and Mediterranean Seas (Table 3 and Table 4).

Therefore, these differences in fatty acid composition could be due to differences in temperature, salinity, pH, and depth between the Maragani River (in the Mediterranean Sea) and the Adriatic Sea [48]. The SFA content was higher in the Adriatic carapace samples, with palmitic acid (C:16) and linoleic acid (C:18) dominating in both samples. The total SFA values reported in this study were higher than those previously reported by other authors [41,49]. The highest MUFAs were found in crab meat from both Seas. The results demonstrated that the MUFA content was consistent with that reported by other authors [43]. Palmitoleic acid was the major MUFA in all blue crab samples, followed by oleic acid. The levels of PUFAs were found to be higher in samples from the Mediterranean Sea than in those from the Adriatic Sea. Moreover, PUFA levels were consistent with other values for blue crab samples from the Mediterranean region but significantly lower for samples from the Adriatic Sea [50]. In addition, a maximum n6/n3 ratio of 250 mg/day is recommended; higher values could be harmful to health and cause cardiovascular disease. In this study, the n6/n3 ratio ranged from 0.19 to 0.22, which was lower than the recommended dose. In addition, the n3/n6 ratio ranged from 4.35 to 5.35, which is higher than the other results for commercial crab samples [51]. Given the higher proportions of EPA and DHA in marine products, FLQ is a suitable supplement. The absolute quantities of EPA and DHA are more important than the relative proportion, so FLQ may be considered a supplement for EPA + DHA. Consequently, other authors evaluated the fatty acid profile using this value, noting that it could differ significantly between closely related species [52]. The FLQ results exhibited a range consistent with those of other crab samples [49]. Table 3 and Table 4 present the atherogenic (AI) and thrombogenic (TI) indices of samples from the Adriatic and Mediterranean Seas, respectively. These values were similar in the crabs from both seas. Moreover, the levels reported were lower than those observed for other crustacean [53] and shrimps [54].

The findings on the overall composition of fatty acids, including MUFAs, PUFAs, SFAs, AI, TI, and FLQ, suggest that blue crab has a good nutritional profile.

The composition of the initial microbiota and SSOs in fresh seafood products is related to the quality of the water in which they live and the hygiene standards adopted in the production and transport chain. Moreover, crustaceans in general and blue crabs in particular are highly perishable fishery products due to their high microbiological and enzymatic activity, which leads to a rapid reduction in their quality. In this regard, SSOs are key players because they can produce metabolites that alter the sensory properties of a product, leading to its rejection by consumers. The determination of initial SSO levels, which in this study proved to be low, and the black colony counts (2.74 ± 1.26 log CFU/g), which were similar to the load of 2.28 ± 0.33 log CFU/g reported by Parlapani et al. [55] in *C. sapidus*, made it possible to hypothesize that the meat of the crabs caught in the study areas is qualitatively suitable [40]. Furthermore, the results showed the absence of potential pathogens such as *L. monocytogenes*, *S. enterica*, *V. parahaemolyticus*, and *V. cholerae*, confirming the good water hygiene quality in the sampling area. However, it is necessary to underline that the small number of crabs examined requires further studies in the future.

With regard to microplastics analysis, although there are currently few studies in the literature concerning microplastics detection in *C. sapidus*, the particle concentrations found in this study were consistent with those observed by Compa et al. [56] and Waddell et al. [57], who reported values of 1.0 ± 1.3 items/individual and 0.87 items/individual, respectively [41,42]. Further studies have found more abundant particle values in *C. sapidus*, as documented in Lesina Lagoon, Italy (2.5 ± 1.6 items/individual), and in the nature reserve lagoonal complex of Kune-Vain, Albania (11.0 ± 1.85 items/individual) [43,44]. The high levels of MP pollution found in crabs in Albania contrast with those found in the nearby Adriatic Sea in this study. This, as the authors hypothesize, could be mainly due to an increase in activities that generate plastic waste and urbanization in the surrounding area. Furthermore, in the Adriatic Sea, higher MP numbers were found in different organisms, especially in mussels, related to the different feeding habits of bivalves compared to crabs [45,46].

Fibers were the only type of particles observed in the study, most of which (n. 6) were cotton, with two polyesters. This is in line with other studies, whereas the fiber diameter (varying between 100 µm to 2000 µm) contrasts with the findings of other authors [42,47,48].

## 5. Conclusions

Blue crabs are a valuable species in their natural habitat, providing food for the local population and income for fishermen. However, in recently invaded areas, such as the Italian coasts, blue crab is considered a threat to and problem for the marine ecosystem. This occurs because it feeds on commercially valuable fish species. The present study presents a comprehensive chemical and microbiological characterization, thereby establishing blue crab as a viable source of human nutrition. Furthermore, the study revealed that the carapace of the blue crab, which is typically regarded as waste due to its inedible nature, can be used as a feed for aquaculture. The heavy-metal data indicate that there is no risk of toxicity associated with consuming this controversial food. The results of the lipid profile analysis also indicate that blue crab can be incorporated into a low-calorie diet. The results of the study did not reveal any difference in the chemical and nutritional composition of blue crab catch between the two different seas. In conclusion, this study suggests that *Callinectes sapidus* is a valuable seafood, and its carapace could be used as a compliant feed for aquaculture due to its metal composition and microbiological content. Consequently, the blue crab can be considered beneficial to human health.

## Figures and Tables

**Table 1:** *Callinectes sapidus* chemical proximate composition.

Sampling Area	Sample Portion	Moisture (%)	Protein (%)	Ash (%)	Total Fat Content (%)
Adriatic Sea	Crab meat	78.22 ± 0.05 ^a^	15.22 ± 0.06 ^a^	1.62 ± 0.05 ^a^	4.47 ± 0.06 ^a^
	Claw meat	74.33 ± 0.06 ^b^	20.24 ± 0.09 ^b^	1.32 ± 0.03 ^b^	3.85 ± 0.05 ^b^
	Carapace	76.31 ± 0.03 ^c^	21.04 ± 0.04 ^c^	0.84 ± 0.01 ^a^	1.02 ± 0.04 ^c^
Mediterranean Sea (Maragani River)	Crab meat	78.16 ± 0.10 ^a^	13.82 ± 0.14 ^d^	1.55 ± 0.03 ^c^	6.21 ± 0.06 ^d^
	Claw meat	74.94 ± 0.07 ^d^	19.52 ± 0.10 ^e^	1.07 ± 0.04 ^b^	4.27 ± 0.05 ^e^
	Carapace	76.90 ± 0.02 ^e^	20.24 ± 0.05 ^b^	0.91 ± 0.01 ^d^	1.13 ± 0.04 ^f^

Data are reported as means ± standard deviations. Different superscript letters in the same column indicate significantly different values (*p* < 0.05 by post-hoc Tukey’s HSD test); same superscript letters in the same column indicate not significantly different values (*p* > 0.05 by post-hoc Tukey’s HSD test).

**Table 2:** Mineral contents of blue crab meat, claw meat, and carapace from the Adriatic and Mediterranean Seas.

	Adriatic Sea			Mediterranean Sea		
Minerals (mg/kg)	Crab Meat	Claw Meat	Carapace	Crab Meat	Claw Meat	Carapace
Al	49.01 ± 6.33 ^a^	28.76 ± 7.16 ^a^	253.21 ± 7.03 ^b^	59.32 ± 4.46 ^a^	32.19 ± 5.26 ^a^	354.44 ± 21.23 ^c^
As	0.07 ± 0.01 ^a^	0.05 ± 0.01 ^a^	n/d	0.10 ± 0.02 ^b^	0.05 ± 0.01 ^a^	n/d
Be	n/d	n/d	n/d	n/d	n/d	n/d
Ca	1586.35 ± 56.83 ^a^	1343.37 ± 26.56 ^b^	3547.01 ± 41.28 ^c^	1555.42 ± 42.07 ^a^	1379.51 ± 20.55 ^b^	3504.38 ± 39.04 ^c^
Cd *	0.11 ± 0.02 ^a^	0.08 ± 0.01 ^b^	n/d	0.13 ± 0.01 ^a^	0.06 ± 0.01 ^b^	n/d
Co	0.10 ± 0.01 ^a^	0.15 ± 0.02 ^a^	2.80 ± 0.19 ^b^	0.12 ± 0.02 ^a^	0.13 ± 0.02 ^a^	3.30 ± 0.14 ^c^
Cr	0.13 ± 0.01 ^a^	0.08 ± 0.01 ^b^	n/d	0.12 ± 0.02 ^b^	0.07 ± 0.01 ^b^	n/d
Cu	12.77 ± 0.48 ^a^	7.24 ± 0.16 ^b^	3.12 ± 0.23 ^c^	11.41 ± 0.34 ^d^	8.45 ± 0.14 ^e^	3.55 ± 0.23 ^f^
Fe	20.45 ± 0.40 ^a^	15.56 ± 0.30 ^b^	50.12 ± 0.28 ^c^	19.51 ± 0.45 ^a^	12.63 ± 0.28 ^d^	55.47 ± 0.49 ^e^
Hg *	0.04 ± 0.01 ^a^	0.06 ± 0.01 ^a^	0.02 ± 0.01 ^b^	0.04 ± 0.01 ^a^	0.11 ± 0.02 ^c^	0.02 ± 0.01 ^b^
Li	n/d	n/d	n/d	n/d	n/d	n/d
Mg	945.67 ± 21.97 ^a^	871.44 ± 16.37 ^b^	1599.85 ± 22.87 ^c^	992.69 ± 8.94 ^a^	854.47 ± 19.39 ^b^	1643.31 ± 22.08 ^c^
Mn	3.67 ± 0.23 ^a^	2.57 ± 0.21 ^a^	10.64 ± 0.43 ^b^	4.71 ± 0.20 ^c^	2.89 ± 0.18 ^d^	9.10 ± 0.12 ^e^
Mo	n/d	n/d	n/d	n/d	n/d	n/d
Na	4107.23 ± 53.96 ^a^	3800.09 ± 25.95 ^b^	4572.72 ± 44.63 ^c^	3984.41 ± 27.48 ^d^	3521.24 ± 53.44 ^e^	4505.31 ± 46.09 ^f^
Ni	0.19 ± 0.02 ^a^	0.12 ± 0.03 ^a^	n/d	0.21 ± 0.04 ^b^	0.16 ± 0.03 ^b^	n/d
P	1724.95 ± 45.55 ^a^	1591.02 ± 29.46 ^b^	2137.77 ± 35.91 ^c^	1793.69 ± 9.79 ^a^	1528.20 ± 38.44 ^b^	2105.09 ± 77.77 ^c^
Pb *	0.21 ± 0.02 ^a^	0.23 ± 0.05 ^a^	n/d	0.19 ± 0.03 ^a^	0.25 ± 0.03 ^a^	n/d
Sb	n/d	n/d	n/d	n/d	n/d	n/d
Se	156.38 ± 5.33 ^a^	144.29 ± 4.43 ^b^	33.23 ± 1.87 ^c^	151.29 ± 1.98 ^a^	149.35 ± 1.31 ^b^	35.66 ± 0.47 ^c^
Ti	n/d	n/d	n/d	n/d	n/d	n/d
V	n/d	n/d	n/d	n/d	n/d	n/d
Zn	86.13 ± 0.98 ^a^	66.57 ± 0.41 ^b^	105.47 ± 0.69 ^c^	88.99 ± 1.42 ^a^	64.87 ± 1.83 ^b^	110.92 ± 0.56 ^d^

Data are reported as means ± standard deviations. Different superscript letters in the same row indicate significantly different values (*p* < 0.05 by post-hoc Tukey’s HSD test); same superscript letters in the same column indicate not significantly different values (*p* > 0.05 by post-hoc Tukey’s 350 HSD test). n/d = not detected (<LOQ). * Toxic metals for crustaceans according to EFSA with their respective limit values: Cd, Pb, and Hg (0.50 mg/kg).

**Table 3:** Fatty acid composition of blue crab meat, claw meat, and carapace from the Adriatic Sea.

Fatty Acid	Crab Meat	Claw Meat	Carapace
C 10:0	0.30 ± 0.04 ^a^	0.18 ± 0.02 ^b^	0.12 ± 0.04 ^b^
C 12:0	0.27 ± 0.03 ^a^	0.07 ± 0.03 ^b^	0.29 ± 0.02 ^a^
C 14:0	0.82 ± 0.03 ^a^	0.76 ± 0.05 ^b^	0.88 ± 0.04 ^a^
C 15:0	0.65 ± 0.03 ^a^	0.52 ± 0.03 ^b^	0.32 ± 0.02 ^c^
C 16:0	14.84 ± 0.04 ^a^	14.73 ± 0.02 ^b^	14.43 ± 0.04 ^c^
C 17:0	1.27 ± 0.05 ^a^	1.11 ± 0.02 ^b^	1.96 ± 0.05 ^c^
C 18:0	10.05 ± 0.04 ^a^	10.03 ± 0.04 ^a^	10.14 ± 0.04 ^b^
C 20:0	0.35 ± 0.05 ^a^	0.53 ± 0.04 ^b^	0.85 ± 0.04 ^c^
C 21:0	0.59 ± 0.03 ^a^	0.62 ± 0.01 ^a^	0.73 ± 0.04 ^b^
C 22:0	0.13 ± 0.03 ^a^	0.09 ± 0.03 ^a^	0.04 ± 0.01 ^b^
C 23:0	0.01 ± 0.00 ^ns^	0.01 ± 0.00 ^ns^	0.01 ± 0.00 ^ns^
C 24:0	0.16 ± 0.04 ^a^	0.08 ± 0.02 ^b^	0.04 ± 0.02 ^b^
∑ SFA	29.42 ± 0.17 ^a^	28.72 ± 0.04 ^b^	29.80 ± 0.02 ^c^
C 14:1	0.01 ± 0.00 ^ns^	0.01 ± 0.00 ^ns^	0.01 ± 0.00 ^ns^
C 15:1	0.23 ± 0.01 ^a^	0.30 ± 0.02 ^a^	0.15 ± 0.04 ^b^
C 16:1 n-9	0.32 ± 0.01 ^a^	0.42 ± 0.02 ^b^	0.54 ± 0.03 ^c^
C 16:1 n-7	7.85 ± 0.04 ^a^	8.73 ± 0.02 ^b^	8.85 ± 0.04 ^c^
C 16:1 n-5	0.29 ± 0.03 ^a^	0.21 ± 0.02 ^b^	0.44 ± 0.04 ^c^
C 17:1	1.25 ± 0.04 ^a^	1.12 ± 0.03 ^b^	1.05 ± 0.02 ^b^
C 18:1 cis n-9	14.54 ± 0.13 ^a^	14.06 ± 0.02 ^b^	14.74 ± 0.04 ^c^
C 18:1 cis vaccenic	3.83 ± 0.04 ^a^	3.43 ± 0.03 ^b^	3.66 ± 0.03 ^c^
C 20:1 n-11	0.74 ± 0.04 ^a^	0.55 ± 0.03 ^b^	0.25 ± 0.04 ^c^
C 20:1 n-9	0.47 ± 0.03 ^a^	0.33 ± 0.03 ^b^	0.69 ± 0.01 ^c^
C 20:1 n-7	0.95 ± 0.02 ^a^	1.01 ± 0.02 ^a^	0.93 ± 0.03 ^a^
C 22:1 n-11	0.51 ± 0.02 ^a^	0.73 ± 0.05 ^b^	0.16 ± 0.03 ^c^
C 22:1 n-9	0.09 ± 0.01 ^a^	0.15 ± 0.01 ^b^	0.12 ± 0.03 ^a^
C 22:1 n-7	0.01 ± 0.00 ^ns^	0.01 ± 0.00 ^ns^	0.01 ± 0.00 ^ns^
∑ MUFA	31.07 ± 0.04 ^a^	31.02 ± 0.02 ^a^	31.59 ± 0.11 ^b^
C 16:3 n-4	0.01 ± 0.00	0.35 ± 0.03 ^a^	0.01 ± 0.00
C 16:4 n-4	0.21 ± 0.03 ^a^	0.01 ± 0.00	0.04 ± 0.02 ^b^
C 18:3 n-3	0.87 ± 0.02 ^a^	0.83 ± 0.02 ^a^	0.76 ± 0.03 ^b^
C 18:4 n-3	0.28 ± 0.02 ^a^	0.31 ± 0.02 ^a^	0.35 ± 0.03 ^b^
C 18:2 cis	2.03 ± 0.02 ^a^	2.24 ± 0.04 ^b^	2.14 ± 0.03 ^c^
C 20:2 n-6	0.23 ± 0.05 ^a^	0.41 ± 0.02 ^b^	0.20 ± 0.02 ^a^
C 20:3 n-6	0.20 ± 0.02 ^a^	0.15 ± 0.03 ^a^	0.05 ± 0.01 ^b^
C 20:4 n-6	5.77 ± 0.03 ^a^	5.68 ± 0.03 ^a^	5.55 ± 0.04 ^b^
C 20:5 n-3	17.12 ± 0.06 ^a^	17.24 ± 0.03 ^b^	17.13 ± 0.03 ^a^
C 21:5 n-3	0.49 ± 0.02 ^a^	0.01 ± 0.00	0.77 ± 0.05 ^b^
C 22:2	0.23 ± 0.02 ^a^	0.35 ± 0.04 ^b^	0.16 ± 0.05 ^a^
C 22:5 n-6	0.38 ± 0.02 ^a^	0.65 ± 0.03 ^b^	0.28 ± 0.02 ^c^
C 22:5 n-3	0.16 ± 0.05 ^a^	0.36 ± 0.02 ^b^	0.07 ± 0.04 ^c^
C 22:6 n-3	11.53 ± 0.03 ^a^	11.66 ± 0.04 ^b^	11.11 ± 0.01 ^c^
∑ PUFA	39.50 ± 0.08 ^a^	40.26 ± 0.08 ^b^	38.61 ± 0.12 ^c^
∑ n3	30.45 ± 0.03 ^a^	30.41 ± 0.03 ^a^	32.32 ± 0.09 ^b^
∑ n6	6.59 ± 0.08 ^a^	6.89 ± 0.03 ^b^	6.08 ± 0.07 ^c^
n6/n3	0.22 ± 0.01 ^a^	0.23 ± 0.01 ^a^	0.19 ± 0.00 ^b^
n3/n6	4.62 ± 0.06 ^a^	4.41 ± 0.02 ^b^	5.32 ± 0.06 ^c^
IA	0.27 ± 0.01 ^a^	0.26 ± 0.01 ^a^	0.26 ± 0.01 ^a^
TI	0.22 ± 0.01 ^a^	0.22 ± 0.00 ^a^	0.21 ± 0.00 ^a^
FLQ	5.59 ± 0.09 ^a^	5.58 ± 0.04 ^a^	6.02 ± 0.03 ^b^

Data are reported as means ± standard deviations. Different letters within a row are significantly different (*p* < 0.05). Different superscript letters in the same row indicate significantly different values (*p* < 0.05 by post-hoc Tukey’s HSD test); same superscript letters in the same column indicate not significantly different values (*p* > 0.05 by post-hoc Tukey’s HSD test). ns = no significant difference (*p* ≥ 0.05).

**Table 4:** Total fat (g/100 g) and fatty acid composition (%) of blue crab meat, claw meat, and carapace from the Mediterranean Sea.

Fatty Acid	Crab Meat	Claw Meat	Carapace
C 10:0	0.12 ± 0.03 ^b^	0.32 ± 0.02 ^a^	0.04 ± 0.02 ^c^
C 11:0	0.01 ± 0.00	0.31 ± 0.03	0.01 ± 0.00
C 12:0	0.17 ± 0.03 ^c^	0.18 ± 0.04 ^d^	0.28 ± 0.02 ^a^
C 14:0	0.52 ± 0.04 ^c^	0.67 ± 0.02 ^b^	0.89 ± 0.03 ^a^
C 15:0	0.57 ± 0.04 ^a^	0.53 ± 0.06 ^b^	0.36 ± 0.03 ^c^
C 16:0	14.86 ± 0.03 ^a^	14.84 ± 0.02 ^a^	14.85 ± 0.04 ^a^
C 17:0	1.18 ± 0.03 ^b^	1.15 ± 0.03 ^b^	1.50 ± 0.01 ^d^
C 18:0	10.37 ± 0.03 ^c^	10.72 ± 0.03 ^d^	10.25 ± 0.03 ^e^
C 20:0	0.30 ± 0.02 ^a^	0.49 ± 0.03 ^b^	0.95 ± 0.05 ^d^
C 21:0	0.22 ± 0.08 ^c^	0.54 ± 0.04 ^a^	0.91 ± 0.02 ^d^
C 22:0	0.10 ± 0.02 ^a^	0.05 ± 0.03 ^b^	0.02 ± 0.01 ^b^
C 23:0	0.01 ± 0.00 ^ns^	0.01 ± 0.00 ^ns^	0.01 ± 0.00 ^ns^
C 24:0	0.13 ± 0.03 ^a^	0.04 ± 0.03 ^b^	0.03 ± 0.02 ^b^
∑ SFA	28.48 ± 0.10 ^b^	29.88 ± 0.12 ^c^	30.09 ± 0.10 ^d^
C 14:1	0.01 ± 0.00 ^ns^	0.01 ± 0.00 ^ns^	0.01 ± 0.00 ^ns^
C 15:1	0.17 ± 0.04 ^a^	0.27 ± 0.04 ^a^	0.10 ± 0.03 ^b^
C 16:1 n-9	0.43 ± 0.01 ^b^	0.36 ± 0.03 ^a^	0.58 ± 0.02 ^c^
C 16:1 n-7	8.34 ± 0.03 ^d^	7.96 ± 0.04 ^e^	8.97 ± 0.02 ^f^
C 16:1 n-5	0.20 ± 0.03 ^d^	0.26 ± 0.01 ^a^	0.28 ± 0.03 ^a^
C 17:1	1.08 ± 0.03 ^b^	1.04 ± 0.04 ^b^	0.93 ± 0.04 ^c^
C 18:1 cis n-9	14.26 ± 0.02 ^d^	14.15 ± 0.02 ^b^	14.46 ± 0.04 ^a^
C 18:1 cis vaccenic	3.92 ± 0.02 ^d^	3.54 ± 0.04 ^e^	3.66 ± 0.03 ^f^
C 20:1 n-11	0.36 ± 0.02 ^d^	0.17 ± 0.02 ^e^	0.27 ± 0.02 ^d^
C 20:1 n-9	0.32 ± 0.03 ^b^	0.25 ± 0.03 ^e^	0.42 ± 0.03 ^a^
C 20:1 n-7	1.04 ± 0.04 ^b^	0.91 ± 0.03 ^c^	0.90 ± 0.02 ^d^
C 22:1 n-11	0.46 ± 0.08 ^a^	0.41 ± 0.02 ^a^	0.23 ± 0.02 ^c^
C 22:1 n-9	0.06 ± 0.03 ^c^	0.14 ± 0.03 ^b^	0.13 ± 0.02 ^b^
C 22:1 n-7	0.01 ± 0.00 ^ns^	0.01 ± 0.00 ^ns^	0.01 ± 0.00 ^ns^
∑ MUFA	30.48 ± 0.21 ^a^	29.46 ± 0.10 ^c^	30.95 ± 0.13 ^a^
C 16:3 n-4	0.01 ± 0.00	0.34 ± 0.06 ^a^	0.01 ± 0.00
C 16:4 n-4	0.18 ± 0.05 ^a^	0.01 ± 0.00	0.12 ± 0.03 ^c^
C 18:3 n-3	0.86 ± 0.03 ^a^	0.86 ± 0.04 ^a^	0.93 ± 0.03 ^a^
C 18:4 n-3	0.23 ± 0.02 ^a^	0.34 ± 0.03 ^b^	0.29 ± 0.02 ^a^
C 18:2 cis	2.84 ± 0.04 ^c^	2.31 ± 0.03 ^b^	2.03 ± 0.04 ^a^
C 20:2 n-6	0.35 ± 0.04 ^b^	0.50 ± 0.02 ^c^	0.36 ± 0.03 ^b^
C 20:3 n-6	0.25 ± 0.04 ^a^	0.21 ± 0.02 ^a^	0.05 ± 0.04 ^b^
C 20:4 n-6	5.63 ± 0.04 ^b^	5.46 ± 0.06 ^b^	5.34 ± 0.03 ^c^
C 20:5 n-3	17.25 ± 0.04 ^b^	17.28 ± 0.02 ^b^	17.35 ± 0.04 ^c^
C 21:5 n-3	0.45 ± 0.04 ^c^	0.01 ± 0.00	0.75 ± 0.04 ^b^
C 22:2	0.13 ± 0.02 ^c^	0.38 ± 0.02 ^b^	0.23 ± 0.02 ^a^
C 22:5 n-6	0.54 ± 0.04 ^d^	0.86 ± 0.04 ^e^	0.32 ± 0.02 ^a^
C 22:5 n-3	0.15 ± 0.04 ^a^	0.15 ± 0.02 ^a^	0.12 ± 0.02 ^a^
C 22:6 n-3	12.17 ± 0.03 ^d^	11.95 ± 0.04 ^e^	11.04 ± 0.03 ^c^
∑ PUFA	41.03 ± 0.13 ^d^	40.66 ± 0.17 ^d^	38.94 ± 0.08 ^e^
∑ n3	31.12 ± 0.06 ^c^	30.58 ± 0.10 ^a^	32.51 ± 0.06 ^d^
∑ n6	6.77 ± 0.08 ^b^	7.04 ± 0.05 ^b^	6.07 ± 0.05 ^d^
n6/n3	0.22 ± 0.01 ^a^	0.23 ± 0.01 ^c^	0.19 ± 0.01 ^b^
n3/n6	4.60 ± 0.06 ^a^	4.35 ± 0.02 ^b^	5.35 ± 0.04 ^c^
IA	0.25 ± 0.01 ^a^	0.26 ± 0.00 ^a^	0.27 ± 0.00 ^a^
TI	0.22 ± 0.00 ^a^	0.23 ± 0.01 ^a^	0.21 ± 0.01 ^a^
FLQ	5.09 ± 0.05 ^c^	5.32 ± 0.05 ^d^	6.31 ± 0.03 ^e^

Data are reported as means ± standard deviations. Different letters within a row are significantly different (*p* < 0.05). Different superscript letters in the same row indicate significantly different values (*p* < 0.05 by post-hoc Tukey’s HSD test); same superscript letters in the same column indicate not significantly different values (*p* > 0.05 by post-hoc Tukey’s HSD test). ns = no significant difference (*p* ≥ 0.05).

## Data Availability

The data presented in this study are available upon request from the corresponding author.
